# The Impacts of Tumor and Tumor Associated Epilepsy on Subcortical Brain Structures and Long Distance Connectivity in Patients With Low Grade Glioma

**DOI:** 10.3389/fneur.2018.01004

**Published:** 2018-11-27

**Authors:** Bibi L. J. Bouwen, Kay J. Pieterman, Marion Smits, Clemens M. F. Dirven, Zhenyu Gao, Arnaud J. P. E. Vincent

**Affiliations:** ^1^Department of Neuroscience, Erasmus MC, University Medical Center Rotterdam, Rotterdam, Netherlands; ^2^Department of Neurosurgery, Erasmus MC, University Medical Center Rotterdam, Rotterdam, Netherlands; ^3^Department of Radiology and Nuclear Medicine, Erasmus MC, University Medical Center Rotterdam, Rotterdam, Netherlands

**Keywords:** glioma, diffusion tensor imaging, connectivity, MRI, tumor associated epilepsy

## Abstract

Low grade gliomas in cerebral cortex often cause symptoms related to higher cerebral functions such as attention, memory and executive function before treatment is initiated. Interestingly, focal tumors residing in one cortical region can lead to a diverse range of symptoms, indicating that the impact of a tumor is extended to multiple brain regions. We hypothesize that the presence of focal glioma in the cerebral cortex leads to alterations of distant subcortical areas and essential white matter tracts. In this study, we analyzed diffusion tensor imaging scans in glioma patients to study the effect of glioma on subcortical gray matter nuclei and long-distance connectivity. We found that the caudate nucleus, putamen and thalamus were affected by cortical glioma, displaying both volumetric and diffusion alterations. The cerebellar cortex contralateral to the tumor side also showed significant volume decrease. Additionally, tractography of the cortico-striatal and cortico-thalamic projections shows similar diffusion alterations. Tumor associated epilepsy might be an important contributing factor to the found alterations. Our findings indeed confirm concurrent structural and connectivity abrasions of brain areas distant from brain tumor, and provide insights into the pathogenesis of diverse neurological symptoms in glioma patients.

## Introduction

Patients with low grade glioma (LGG) can have a long pre-clinical period with various subclinical neurological symptoms. Tumor associated epilepsy is the most frequent presenting symptom (1) and the occurrence of seizures often leads to early diagnosis. Less acute manifestations such as cognitive or behavioral problems, headaches, and small motor or sensory limitations are also common and can exist for long periods of time before diagnosis is made. Recent studies show that cognitive impairment in glioma patients is already present in the majority of patients before initiation of treatment, suggesting a role for glioma as an important cause of neuro-cognitive deficits (2–4). Epilepsy and other cognitive symptoms affecting higher cerebral functions such as attention, memory, communication, and executive functions can severely affect patients' daily lives, including their neuropsychological wellbeing (5). With median survival ranging from 5 to 15 years, depending on many factors like the histological subtype, patient age, initial tumor size and tumor growth rate (6–11), LGG patients live for many years after treatment and cognitive functioning is one of the key components of their quality of life (12).

Interestingly, the diversity of these symptoms is often seemingly unrelated to tumor location and can occur months or years before diagnosis and remain present after tumor removal (2). These observations indicate that the impact of a focal tumor can extend into multiple functional brain regions. It is therefore possible that the presence of focal glioma leads to alterations of distant subcortical areas and essential white matter (WM) tracts. Current evidence indicates that LGG affects the local neuronal circuits, causing morphological changes in the peri-tumoral cortex and evoking a large immune response leading to altered cell functioning in the peri-tumoral micro-environment (13). Beyond the local environment, much less is known about the effects of glioma on long-distance connectivity and subcortical structures. LGG is a slow developing process evolving over the course of months to years (14–16), providing a large time-frame for subsequent structural alterations in distant areas. Most often, tumors are found in the (sub-) cortical regions of the frontal and temporal lobes, followed by insular, parietal and occipital tumors (17–20). The functional cortical areas of the frontal and temporal cortex (but also parietal and occipital cortex) are strongly and reciprocally connected to specific regions of the nuclei in the basal ganglia and thalami via distinct cortico-striatal and cortico-thalamic pathways (21, 22). These pathways are essential for integration and processing of many types of cerebral inputs including neuro-cognitive functions (21–24). Whether these connections are specifically altered in LGG patients remains to be shown.

Seizures, either focal or generalized tonic-clonic are observed in up to 80% of LGG patients (20, 25, 26). Apart from being a presenting symptom, the recurrence of tumor associated epilepsy after surgery and subsequent therapies can also be indicative of tumor progression (25). Studies in temporal lobe epilepsy demonstrate the presence of diffusion alterations, volumetric changes, and network abnormalities both ipsilateral and contralateral to the epileptic focus. These alterations are found both inside the temporal lobe and in extra-temporal structures including the basal ganglia and thalamus and even extending into the cerebellum (27–29). A recent study also linked the degree and spread of WM abnormalities in temporal lobe epilepsy with mesial temporal sclerosis to the occurrence of neurocognitive deficits (30). Whether the tumor associated epilepsy (TAE) has any relationship with long-distance connectivity changes in LGG patients is totally unknown.

Diffusion-weighted imaging (DWI) is an MRI sequence that can be used to measure diffusivity of water molecules in neuronal tissue. By mapping directional motion of water molecules within brain tissue, DWI allows quantification of brain microstructure such as myelination and axonal density (31), as well as visualization of white matter connectivity (32, 33). In the presence of a malignant process in a (sub-)cortical area, WM tracts, including the cortico-striatal and cortico-thalamic tracts, are at risk to become affected by the growing tumor and the peri-tumoral cortical micro-environment, leading to damaging of these essential pathways (34, 35). Therefore, studying the structural alterations caused by glioma on long-range connections using DWI could lead to more insight into the anatomical substrates of the observed alterations in neurocognitive functioning.

For this study, we used a database of DWI scans of low grade glioma patients undergoing awake surgery for LGG in eloquent cortical regions. Using the conventional T1-weighted sequences and the diffusion-weighted scans we applied an atlas-based approach and generated segmentations of the cortex, subcortical nuclei, and cerebellum. To assess long distance alterations, we analyzed the volumes and diffusion parameters of the subcortical gray matter nuclei and the cerebellum. Additionally, we performed tractography analysis on the reciprocal cortico-striatal and cortico-thalamic WM projections. We found alterations in the subcortical nuclei, contralateral cerebellar hemisphere and WM tracts ipsilateral to the hemisphere containing the tumor. Additionally, we found a clear correlation of tumor associated epilepsy on the found alterations. To our knowledge, this is the first study to assess long distance effects and connectivity changes in subcortical nuclei and cerebellum in a large cohort of LGG patients.

## Materials and methods

### Patients

This study was performed using an MRI database of 106 patients with LGG, scanned between 2006 and 2014 in preparation of an awake craniotomy at Erasmus Medical Centre Rotterdam, The Netherlands. Exclusion criteria were: insufficient data quality of both DWI and T1-weighted scans (visually inspected by 2 independent reviewers in 3 orthogonal planes), radiotherapy or previous brain surgery prior to scanning, complications of LGG that were thought to affect the accuracy of tumor delineation and brain segmentation such as hydrocephalus or extensive perifocal edema. Based on these exclusion criteria, 82 patients were included for further analysis in this study.

Pre-surgical work up consists of clinical evaluation by a neurosurgeon and by an anesthesiologist. Before awake surgery patients are tested and prepared by a neuro-linguist who is also present during the surgery. Selection of patients for awake craniotomy is based on tumor location and grade based on the evaluation of the neurosurgeon and a neuro-radiologist. Tumors growing into eloquent cortical areas including but not limited to the primary motor and sensory cortices, insula, areas of Broca and Wernicke, and the primary visual cortex are resected with an awake surgery to map out and avoid resection of eloquent regions during surgery. Data on patient information, tumor location, histological tumor type, and occurrence of epilepsy was obtained retrospectively. Data on epileptic events was taken from notes from the referring neurologist and the neurosurgeon with history of presenting symptoms, seizure-like episodes and seizure progression or attenuation during course of the disease. Patients were classified as having TAE if they presented with epilepsy as a first symptom, or developed epileptic seizures at any time point before the surgery.

### MRI data acquisition and analysis

All patients underwent standard pre-surgical structural MRI (T1 and T2), mostly with additional DWI scans in the Erasmus Medical Centre Rotterdam between 2006 and 2014 on a 3T scanner. DWI data were acquired using a single-shot spin-echo echo-planar imaging sequence at 3.0 T (GE Healthcare) with an 8-channel head coil. In general, 25 noncollinear gradient directions at *b* = 1,000 s/mm^2^ and 3 images at *b* = 0 s/mm^2^ were acquired. The slice thickness was 2.0–3.5 mm, and in-plane resolution was 1.9–3.4 mm^2^. Raw diffusion MRI data were transferred to an offline workstation.

To allow further analysis, T1-weighted scans were evaluated and scored based on invasion of or close proximity of tumor to the basal ganglia and thalamus independently by two authors. To assess the long distance effects of glioma on deep gray matter nuclei, thalamus and cerebellum, only scans with tumors located outside deep gray matter (DGM) and thalamus were assessed for volumetric and diffusion alterations. Any scans with direct ingrowth into the deep gray matter were excluded, similar for scans with edema or large displacement due to tumor reaching into the DGM and thalamus area. Scans of patients with tumors located outside subcortical nuclei and without visual edema affecting these structures were included, and tumors were semi-automatically delineated using ITK snap version 3.2, with manual adjustment *post hoc*. Tumor segmentations were normalized and registered to MNI space to summarize tumor localization in our cohort. Using the whole brain segmentations, separate masks for three main subcortical nuclei caudate nucleus, putamen and thalamus were made and analyzed. For cerebellum the WM and the cerebellar cortex were segmented.

Preprocessing of DWI data was performed prior to analysis and included visual inspection of raw diffusion MRI data in three orthogonal planes. Subsequently, diffusion data were corrected for motion-induced distortions, Eddy currents and EPI-induced distortions using Explore DTI version 4.8.5 running in Matlab (R2015 The MathWorks Inc., Natick, MA, United States) (36). EPI induced deformities of the diffusion data were corrected using the T1-weighted images as a reference, and correction for motion-induced outliers was robustly performed using the REKINDLE approach, consisting of automatic rejection of outlier-corrupted slices (37, 38). T1-weighted and DWI data were resampled to 1 mm isotropic resolution and spatially aligned, followed by automatic segmentation of T1-weighted images in cortical gray matter and subcortical gray matter structures. Segmentation was performed using FSL and Freesurfer (39–41). Segmented T1-weighted images were subsequently imported in ExploreDTI to perform a ROI-based analysis based on these segmentations. Parameters collected for each anatomical structure were: volume, fractional anisotropy (FA) and mean diffusivity (MD). Impact of tumors on the following structures was assessed, using contralateral brain areas as internal reference: caudate nucleus, putamen, thalamus and cerebellum. For the cases from the HCP database, we performed the same segmentation procedures as mentioned above.

### Tractography analysis

For all DWI images of patients with tumors located peripheral from DGM structures and thalamus, whole brain tractography was performed using a deterministic tractography algorithm. Whole brain tractography was performed using a deterministic tractography approach according to the following settings: seedpoint resolution 1 mm isotropic; seed FA threshold 0.1; tracking threshold range 0.1–1; angle threshold 30; step size 1; fiber length range 50–500 mm. Following whole brain tractography, subcortical structures of interest were used as ROI templates to filter tracts passing through these regions out of whole brain tractography data. Tumor-side and contralateral side WM tracts of subcortical nuclei were compared in individual patients to evaluate the influence of tumor presence on WM connections between cortex and the specific nuclei.

### Statistical analysis

To analyze the effect of tumor associated epilepsy on subcortical nuclei and cerebellum, we compared cases with tumor associated epilepsy to glioma cases with no history of epilepsy before or at the time of the scan. Statistical analysis was performed using SPSS (IBM SPSS statistics 20 Software). Multiple comparison corrections were performed when analyzing the subcortical nuclei, *p* < 0.02 considered significant.

## Results

### Clinical information

**Figure 1:**
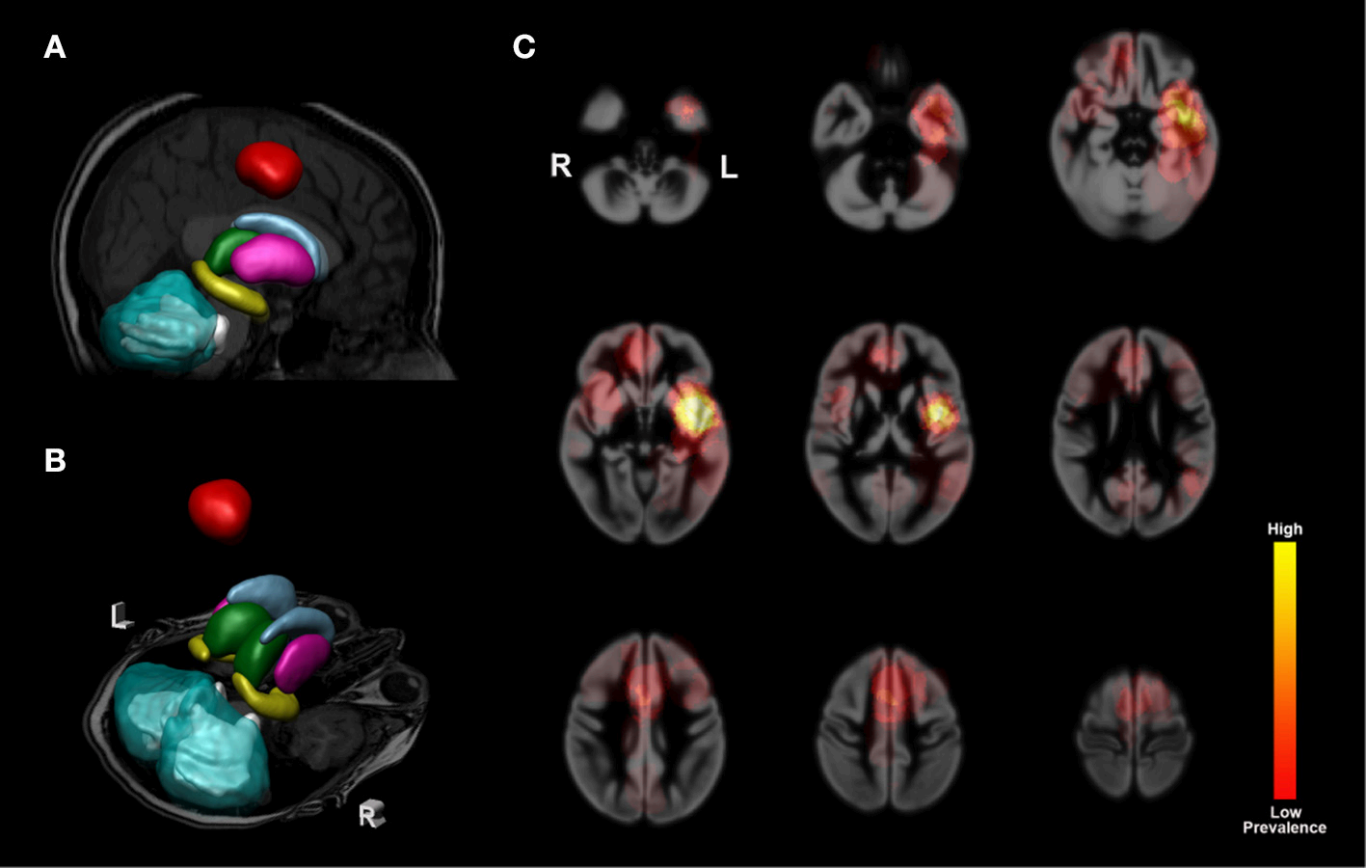
Subcortical nuclei and cerebellum segmentations and population heat map. 3D example images of segmentations projected on a T1 image from a patient. Segmentations used as ROIs for calculations of volume and diffusion values. **(A)** Mid-sagittal view with the left nuclei and left-sided tumor projected onto the T1 image. Tumor segmentation is shown in red. Legend for segmented nuclei: light blue = caudate nuclei, pink = putamen, green = thalamic nuclei, yellow = hippocampus, aquamarine blue = cerebellar cortex, light gray = cerebellar white matter. **(B)** a transverse section at the level of the temporal poles and cerebellum with right and left-sided subcortical nuclei and cerebellar hemispheres shown. Left and right are indicated with L and R, respectively. **(C)** nine transverse sections with a heat map composed of all tumors used in our analysis projected onto a smoothed cortical template image. Higher prevalence is shown in bright yellow, indicating a high number of tumors affecting the cortical region. Color key for heat map shown on the far right.

In total 82 patients were included in this study (for detailed patient information see Table 1). The mean age of the cohort was 41.1 years (range 19–74) with 62.2% male patients. Tumors located in the left hemisphere were more frequent than right sided tumors as a result of the selection criteria for awake surgery. Most tumors were situated in the frontal, fronto-parietal and temporal regions (Figure 1). Approximately equal numbers of oligodendrocytic and astrocytic tumors were present. Fifty-eight scans were assessed as a tumor location not in close proximity to the deep gray matter or thalamus. In a small number of scans either segmentation of T1 data or processing of DWI data failed due to insufficient quality of the data. In these cases, subsets of data (T1 or DWI) were not further analyzed. Precise numbers of scans used are therefore separately mentioned in the results for T1-based ROI analyses and tractography.

**Table 1:** Clinical information.

			**N**	**%**
Patients		Total number	82	100
Sex		Male	51	62.2
		Female	31	37.8
Mean age		Years	41.1
		Range	19–74
Tumor	Side	Left	58	70.7
		Right	24	29.3
	Lobe	Frontal	34	41.4
		Frontotemporal	7	8.6
		Temporal	14	17.1
		Frontoparietal	14	17.1
		Parietal	12	14.6
		Occipital	1	1.2
	Grade	2	55	67.1
		3	27	32.9
	Type	Oligodendroglioma	25	30.5
		Astrocytoma	21	25.6
		Mixed oligoastrocytoma	11	13.4
		Anaplastic oligodendroglioma	11	13.4
		Anaplastic astrocytoma	14	17.1
	Close to basal ganglia	Yes No	24 58	29.3 70.7
Epilepsy	Diagnosis	Yes	57	69.5
		No	25	30.5
	Type	Focal	28	34.1
		Generalized	29	35.4
	Frequency	Once at diagnosis or in surgery	16	19.6
		Yearly-monthly	23	28
		Weekly-daily	12	14.6
		Postoperative seizures/unclear frequency	6	7.3
		No epilepsy	25	30.5
Control	Presenting symptom	Speech problems	3	12
		Incidental finding	12	48
		Change in behavior	3	12
		Headache	6	24
		Palsy	1	4

*Clinical information of all cases included in the initial database. Total numbers and percentages given. N, numbers of patients*.

### Volume alterations in subcortical nuclei and cerebellum of LGG patients

**Figure 2:**
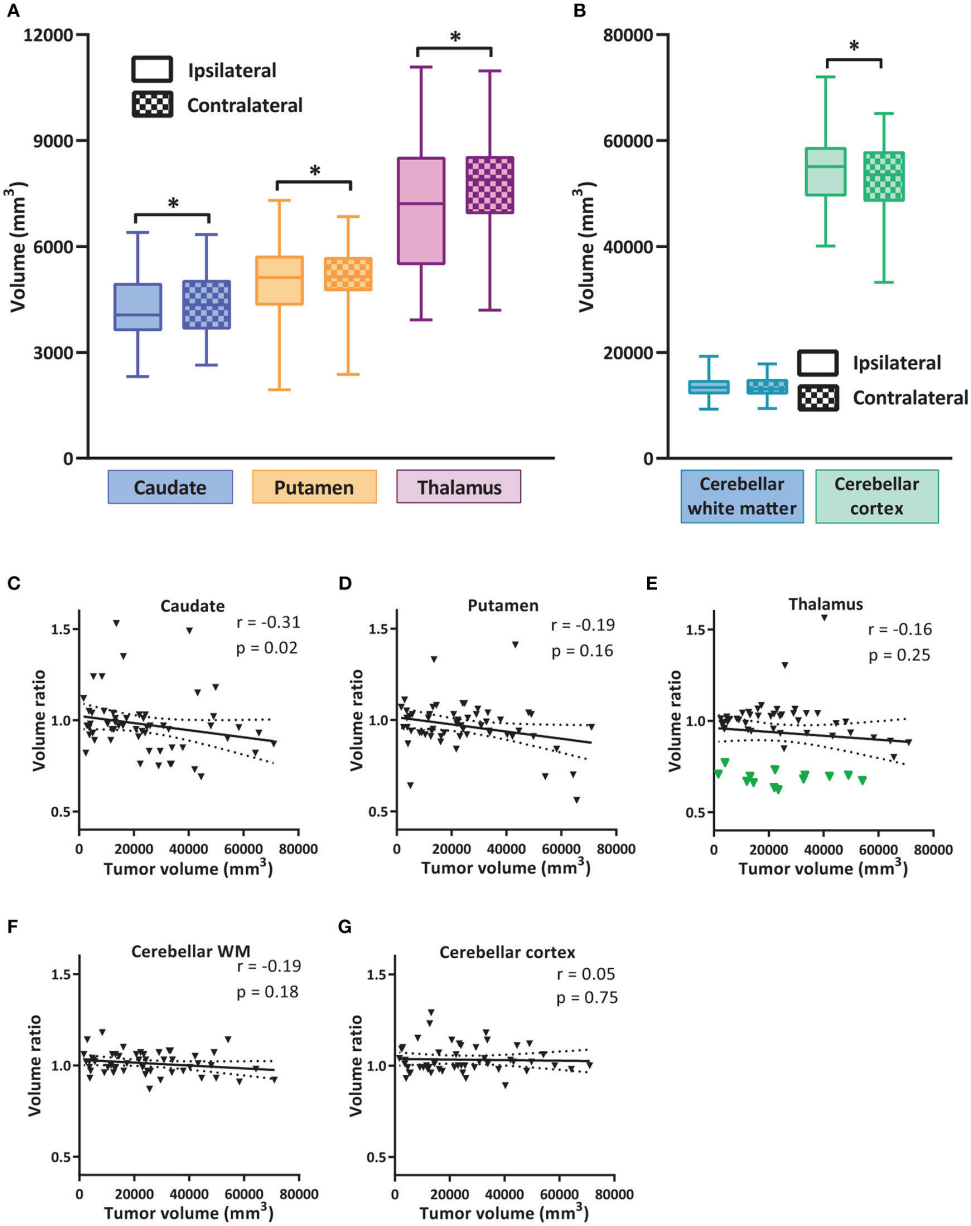
Volume alterations of subcortical nuclei and cerebellum. **(A)** Volumes of subcortical nuclei caudate, putamen and thalamus ipsilateral and contralateral to tumor hemisphere. Box and whiskers are given, showing the median and 25–75% percentile with whiskers for the minimum and maximum. The ipsilateral hemisphere data is shown in unchecked boxes, checked boxes are contralateral hemisphere. Volume is given in mm^3^. In **(B)** the volumes for cerebellar WM and cortex are shown. Wilcoxon matched-pairs signed rank tests were used per structure. Asterisks indicate significant results. **(C–G)** Correlation of tumor volume vs. the volume ratio (ipsilateral volume divided by contralateral volume) of subcortical nuclei and cerebellar sub regions. Green triangles in **(E)** indicate group with volume ratio < 0.8. Linear regression line with 95% confidence interval shown. Spearman *r* and *p*-value are given. *P ≤ 0.02.

We first examined the impacts of focal cortical tumor on the size of subcortical structures. For this study, we focused on three main subcortical nuclei (caudate nucleus, putamen and thalamus), as well as the cerebellar WM and cortex. The volumes of nuclei ipsilateral from the tumor were compared to the contralateral side of the same patient. The volumes of the caudate, putamen, and thalamus ipsilateral to the tumor side were significantly decreased (Figure 2A and Table 2). In the cerebellum, the cerebellar cortex contralateral to the tumor side showed a significant reduction of volume compared to the ipsilateral side, whereas the size of cerebellar WM was comparable (Figure 2B).

**Table 2:** Volumes alterations in subcortical nuclei and cerebellum.

**Structure**	**Ipsilateral volume (mm^3^)**	**Contralateral volume (mm^3^)**	***P***	**Pairing spearman r**	***P***
Caudate	4,209 ± 951	4,346 ± 871	0.02	0.74	<0.001
Putamen	4,965 ± 1,058	5,135 ± 878	0.02	0.81	<0.001
Thalamus	7,067 ± 1,838	7,583 ± 1,520	0.01	0.81	<0.001
Cerebellar WM	13,549 ± 1,818	13,388 ± 1,750	0.26	0.88	<0.001
Cerebellar cortex	54,671 ± 6,617	53,052 ± 6,575	0.02	0.85	<0.001

*Mean volumes ± standard deviation for ipsilateral hemisphere in relation to the tumor and the contralateral hemisphere, n = 57 pairs for DGM, n = 53 pairs for cerebellum. Wilcoxon matched-pairs signed rank test was used. For each structure the pairing Spearman r with significance is given. WM, white matter*.

Although we strictly selected the patients in which tumor did not invade the subcortical structures, we could not exclude the possibility that tumor physically compressed the surrounding tissue, leading to a reduction in size of subcortical nuclei. To analyze the effect of tumor size on the volume alterations of nuclei, we determined the correlation between tumor size and the change in the ratio of the volume ipsi- vs. contralateral for the subcortical structures and the cerebellar regions (Figures 2C–G). The volume ratios for the putamen and thalamus did not correlate with the tumor size. Analysis of caudate nucleus showed a small negative correlation with the size of tumor. Additionally, the cerebellar WM and cortex did not show any significant correlation with tumor volume. Interestingly, a subgroup of patients had especially low ratio in ipsilateral vs. contralateral thalamic volumes (Figure 2E, green group, ipsi-contra ratio < 0.8, *N* = 13, see Supplementary Figure 1 for tumor heat maps of this group compared to the rest of the cohort). This ratio had no correlation with the tumor size of those patients. Further exploration showed that this subgroup consisted of left-sided tumors located mainly in the frontal or fronto-parietal regions. Possibly this specific tumor location severely affected the projections to and from somatosensory and supplementary motor areas. Thus, clear reductions in the volumes of subcortical areas ipsilateral to tumor were observed in some LGG patients, which is unlikely to be merely caused by physical compression by the cortical tumor.

### Diffusion alterations in subcortical nuclei and cerebellum of LGG patients

**Figure 3:**
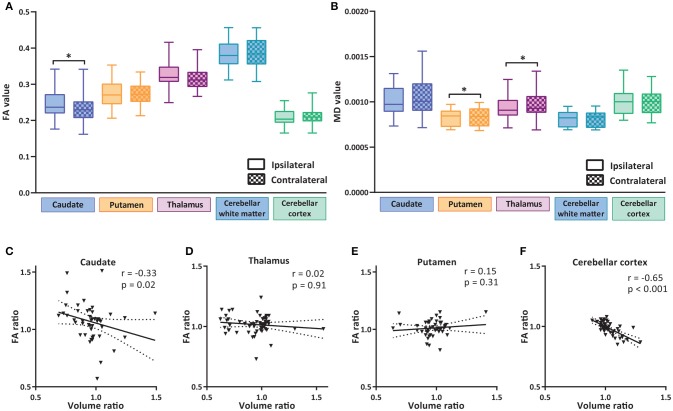
Diffusion alterations in subcortical nuclei and cerebellum. **(A)** Fractional anisotropy (FA) values of subcortical nuclei and cerebellar regions ipsilateral and contralateral to tumor hemisphere. **(B)** Mean diffusivity (MD) values of subcortical nuclei and cerebellar regions ipsi- and contralateral to the tumor. Box and whiskers are given, showing the median and 25–75% percentile with whiskers for the minimum and maximum. The ipsilateral hemisphere data is shown in unchecked boxes, checked boxes are contralateral hemisphere. FA and MD are in arbitrary units. Wilcoxon matched-pairs signed rank tests were used per structure. Asterisks indicate significant results. **(C–F)** Correlation plots of volume ratios (volume ipsilateral divided by volume contralateral) vs. FA ratios (FA ipsilateral divided by FA contralateral) of a specific region. Linear regression line with 95% confidence interval shown. Spearman *r* and *p*-value are given. *P ≤ 0.02.

We next examined whether the reduction in volume of subcortical nuclei and cerebellum was accompanied by structural alterations in these regions by determining fractional anisotropy (FA) and mean diffusivity (MD) of these regions. The caudate nucleus had a significantly increased FA value in the side ipsilateral to the tumor (Figure 3A and Table 3). This increase in FA value negatively correlated with the volume of caudate nucleus (Figure 3C), i.e., the patients with higher FA in the ipsilateral side also had a volume loss on the same side. For thalamus a similar trend was observed, although not significant. For the putamen no significant correlation between FA ratio and volume ratio was observed. In the cerebellum, although no significant differences were observed between ipsi- and contralateral hemisphere in FA values (Figure 3A), a significant negative correlation was observed between volume ratio and FA ratio of ipsi- vs. contralateral cerebellar cortex (Figure 3F). A decrease in mean diffusivity (MD) was observed in the putamen and thalamus ipsilateral to the tumor, but not in caudate and cerebellar regions (Figure 3B).

**Table 3:** Diffusion values in subcortical nuclei and cerebellum.

**Structure**	**FA ipsilateral**	**FA contralateral**	***P***	**Pairing spearman r**	***P***
Caudate	0.247 ± 0.038	0.234 ± 0.036	0.003	0.43	0.001
Putamen	0.274 ± 0.032	0.271 ± 0.029	0.18	0.84	<0.001
Thalamus	0.324 ± 0.036	0.318 ± 0.031	0.04	0.69	<0.001
Cerebellar WM	0.382 ± 0.038	0.383 ± 0.041	0.70	0.92	<0.001
Cerebellar cortex	0.206 ± 0.020	0.210 ± 0.019	0.08	0.76	<0.001
**Structure**	**MD ipsilateral**	**MD contralateral**	***P***	**Pairing spearman r**	***P***
Caudate	0.00101 ± 0.00015	0.00104 ± 0.00018	0.04	0.58	<0.001
Putamen	0.00082 ± 9.0*e*−5	0.00083 ± 9.9*e*−5	0.006	0.92	<0.001
Thalamus	0.00094 ± 0.00012	0.00098 ± 0.00014	<0.001	0.72	<0.001
Cerebellar WM	0.00081 ± 8.57*e*−5	0.00081 ± 8.31*e*−5	0.70	0.92	<0.001
Cerebellar cortex	0.0010 ± 0.00014	0.0010 ± 0.00012	0.19	0.95	<0.001

*Mean values ± standard deviation for ipsilateral hemisphere in relation to the tumor and the contralateral hemisphere, n = 47 pairs for DGM, n = 46 pairs for cerebellum. Wilcoxon matched-pairs signed rank test was used. For each structure the pairing Spearman r with significance is given. WM, white matter*.

### Alterations of WM tracts connecting the subcortical nuclei and cortex

**Figure 4:**
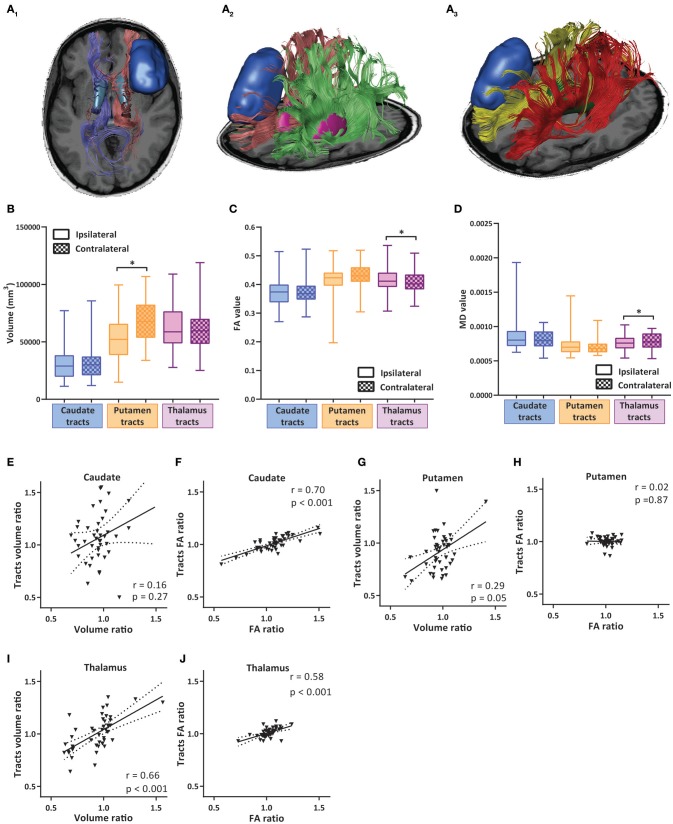
Volumetric and diffusion alterations of cortico-striatal and cortico-thalamic projections. **(A**_1−3_**)** Example images of tractography analysis of cortico-striatal and cortico-thalamic white matter tracts. **(A**_1_**)** Reciprocal projections from caudate nucleus to cortex, transverse view. Tumor shown in dark blue, caudate nuclei in light blue, projections shown in red and purple. **(A**_2_**)** Reciprocal projections from putamen to cortex, tilted transverse view. Tumor shown in dark blue, putamen in pink, projections show in red and green. **(A**_3_**)** Reciprocal projections from thalamic nuclei to cortex, tilted transverse view. Tumor shown in dark blue, thalamic nuclei in green, projections in yellow and red. **(B)** Total volumes of white matter tract bundles to caudate, putamen and thalamus ipsilateral and contralateral to tumor hemisphere. Box and whiskers are given, showing the median and 25–75% percentile with whiskers for the minimum and maximum. The ipsilateral hemisphere data is shown in unchecked boxes, checked boxes are contralateral hemisphere. Volume is given in mm^3^. **(C,D)** FA and MD values of white matter tract bundles to caudate, putamen and thalamus ipsilateral and contralateral to tumor hemisphere. Wilcoxon matched-pairs signed rank tests were used per structure. Asterisks indicate significant results. **(E,G,I)** Correlation plots of volume ratios of respective nuclei (ipsilateral volume divided by contralateral volume) vs. volume ratio of white matter tracts from nucleus to cortex. **(F,H,J)** Correlation plots of FA ratios (ipsilateral FA divided by contralateral FA) of respective nuclei vs. FA ratios of white matter tracts from nucleus to cortex. Linear regression line with 95% confidence interval shown. Spearman *r* and *p*-value are given. *P ≤ 0.02.

Due to the apparent changes in volume and the alterations in diffusion parameters in the subcortical nuclei distant from tumor, we reasoned that one of the factors contributing to these long-distance effects could be altered connections between tumor bearing cortex and the affected subcortical nuclei. Therefore, we performed tractography analysis using the segmentations of the caudate, putamen and thalamus as seed points for tractography of WM tracts connecting the respective nuclei to the cortex (Figure 4A). The volume of the tracts ipsilateral and contralateral to the tumor was not significantly different for the caudate nucleus and thalamus, whereas the volume of the tracts to/from putamen was significantly lower in the ipsilateral side (Figure 4B, see also Table 4). We next analyzed the correlations between the volume of subcortical nuclei and the volume of the tracts project to these nuclei. Positive trends of correlation for all three nuclei were observed, indicating that a decline in ipsilateral volume of the nucleus is associated with a decline in tract size to the nucleus. For putamen and thalamus this relationship was statistically significant (Figures 4E,G,I).

**Table 4:** Volumes of cortico-striatal and cortico-thalamic white matter tracts.

**Cortical WM tracts to/from**	**Ipsilateral volume (mm^3^)**	**Contralateral volume (mm^3^)**	***P***	**Pairing spearman r**	***P***
Caudate	31,157 ± 13,745	30,522 ± 12,989	0.14	0.64	<0.001
Putamen	52,780 ±18,724	67,821 ± 18,022	<0.001	0.39	<0.001
Thalamus	61,734 ± 18,383	60,408 ± 16,553	0.27	0.84	<0.001

*Mean volumes ± standard deviation for ipsilateral hemisphere in relation to the tumor and the contralateral hemisphere, n = 67 pairs for tracts to DGM. Wilcoxon matched-pairs signed rank test was used. For each structure the pairing Spearman r with significance is given. WM, white matter*.

The tracts to the nuclei also showed an increase in the FA value in the cortico-thalamic/thalamo-cortical pathway (Table 5 and Figure 4C). The connections to/from caudate and putamen did not display a significant change in FA values. The ipsi-contra FA ratios for both caudate and thalamus tracts positively correlated with the FA ratios of their targeting subcortical nuclei (Figures 4F,J). No correlation between the FA ratio of putamen tracts and the putamen region was found (Figure 4H). The MD value of the tracts to thalamus was also significantly decreased, but no change was observed in the tracts to the caudate and putamen (Figure 4D). The observed alterations in volume, FA and MD of the long distance tracts to/from putamen and thalamus raise the possibility of a more elaborate influence of LGG on large brain networks rather than mere local effects in the (sub-)cortical region of the tumor.

**Table 5:** Diffusion alterations in cortico-striatal and cortico-thalamic white matter tracts.

**Cortical WM tracts to/from**	**FA ipsilateral**	**FA contralateral**	***P***	**Pairing spearman r**	***P***
Caudate	0.372 ± 0.048	0.375 ± 0.043	0.76	0.45	0.001
Putamen	0.410 ± 0.069	0.432 ± 0.041	0.03	0.48	0.001
Thalamus	0.417 ± 0.039	0.409 ± 0.038	0.02	0.71	<0.001
**Cortical WM tracts to/from**	**MD ipsilateral**	**MD contralateral**	***P***	**Pairing spearman r**	***P***
Caudate	0.00085 ± 0.0002	0.00082 ± 0.00012	0.40	0.70	<0.001
Putamen	0.00075 ± 0.00018	0.00069 ± 7.95*e*−5	0.03	0.68	<0.001
Thalamus	0.00076 ± 9.45*e*−5	0.00079 ± 0.0001	0.003	0.79	<0.001

*Mean values ± standard deviation for ipsilateral hemisphere in relation to the tumor and the contralateral hemisphere, n = 67 pairs for tracts to DGM. Wilcoxon matched-pairs signed rank test was used. For each structure the pairing Spearman r with significance is given. WM, white matter*.

### The occurrence of tumor associated epilepsy correlates with volumetric and diffusion alterations in the subcortical nuclei and cerebellum

**Figure 5:**
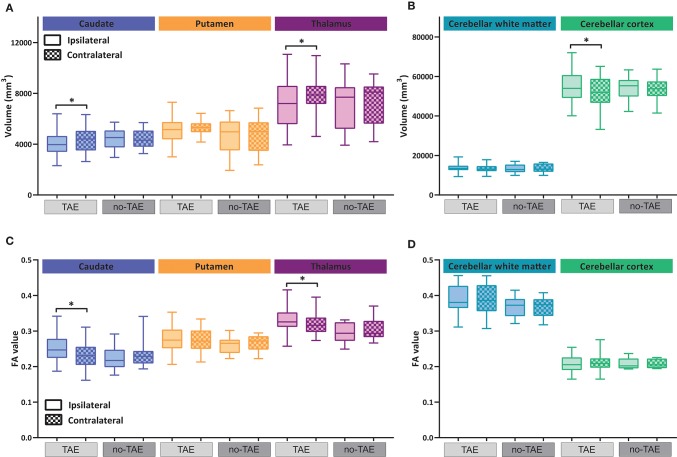
Volumetric and diffusion alterations of subcortical nuclei and cerebellum in TAE and no-TAE patients. **(A,B)** Volumes of subcortical nuclei and cerebellar sub-regions ipsilateral and contralateral to tumor hemisphere. Total group is separated into cases with tumor associated epilepsy (TAE) and no-TAE cases, shown in light gray and dark gray respectively. Box plots showing the median and 25–75% percentile with whiskers for the minimum and maximum. The ipsilateral hemisphere data is shown in unchecked boxes, checked boxes are contralateral hemisphere. **(C,D)** FA values of subcortical nuclei and cerebellar sub-regions ipsilateral and contralateral to tumor hemisphere, again separated into TAE and no-TAE groups. Wilcoxon matched-pairs signed rank tests were used per structure. Asterisks indicate significant results. *P ≤ 0.02.

LGGs located in eloquent brain regions are associated with a high risk of TAE (42). Indeed, a high percentage of patients with TAE pre-operatively were present in our cohort (Table 1). We thus sought to examine the relationship between long distance structural change and TAE. The volumes for the three subcortical nuclei and cerebellum were separately analyzed in patients with and without TAE. The volumes of caudate nucleus and thalamus ipsilateral to the tumor were significantly decreased in the TAE patients (Figure 5A and Table 6), but not in the no-TAE patients. Similarly, the volume of the cerebellar cortex contralateral to the tumor side was decreased only in the TAE patient group (Figure 5B). The location of tumors cannot account for the difference observed between TAE and no-TAE patients. We found wide spread in tumor locations in both patient groups, and no clear difference in the tumor location was found in TAE and no-TAE patients (Supplementary Figure 2). A contributing factor to the observed difference between TAE and no-TAE patients could be a difference in tumor size between groups. We analyzed the tumor size in these two groups of patients. The no-TAE group had a smaller average tumor size than the TAE group. However, no clear correlations between the tumor size and the ipsi/contra volume ratio of thalamus and putamen were observed in TAE and no-TAE groups (Supplementary Figure 3). A significant relation between tumor size and volume ratio was found in caudate for TAE patients, which suggests a direct influence of tumor size on caudate size. It is worth mentioning that patients with severely reduced thalamus volume ratio (ipsi/contra ratio < 0.8, all TAE patients) had extremely diverse tumor sizes, indicating that there is no direct relation between tumor volume and the reduction of thalamus volume ratio in TAE patients.

**Table 6:** Volume alterations in TAE and no-TAE groups in subcortical nuclei and cerebellum.

**Structure**		**Ipsilateral volume (mm^3^)**	**Contralateral volume (mm^3^)**	***P***	**Pairing spearman r**	***P***
Caudate	TAE	4,106 ± 1,031	4,311 ± 937	0.02	0.79	<0.001
Caudate	no-TAE	4,415 ± 1,031	4,415 ± 740	0.37	0.61	<0.001
Putamen	TAE	5,121 ± 859	5,306 ± 542	0.03	0.71	<0.001
Putamen	no-TAE	4,654 ± 1,352	4,793 ± 1,268	0.28	0.90	<0.001
Thalamus	TAE	7,095 ± 1,801	7,780 ± 1,389	0.01	0.72	<0.001
Thalamus	no-TAE	7,012 ± 1,959	7,187 ± 1,723	0.35	0.96	<0.001
Cerebellar WM	TAE	13,705 ± 1,730	13,348 ± 1,682	0.03	0.83	<0.001
Cerebellar WM	no-TAE	13,187 ± 2,017	13,482 ± 1,954	0.12	0.94	<0.001
Cerebellar cortex	TAE	54,812 ± 7,066	52,762 ± 7,135	0.01	0.86	<0.001
Cerebellar cortex	no-TAE	54,345 ± 5,637	53,721 ± 5,197	0.78	0.86	<0.001

*Mean volumes ± standard deviation for ipsilateral hemisphere in relation to the tumor and the contralateral hemisphere, for tumor associated epilepsy (TAE) group n = 38 pairs, for no-TAE group n = 19 pairs. For cerebellum: TAE n = 37 pairs, no-TAE group n = 16 pairs. Wilcoxon matched-pairs signed rank test. For each structure the pairing Spearman r with significance is given. WM, white matter; TAE, tumor associated epilepsy*.

**Figure 6:**
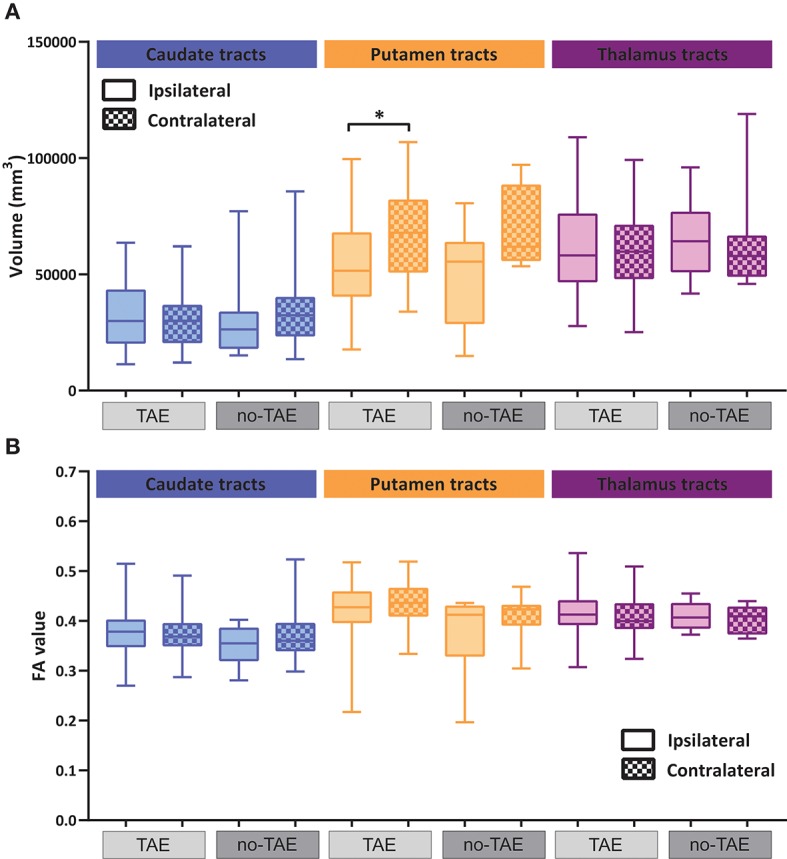
Volumetric and diffusion alterations of cortico-striatal and cortico-thalamic projections in TAE and no-TAE patients. **(A)** Volumes of reciprocal cortico-striatal and cortico-thalamic projections ipsilateral and contralateral to tumor hemisphere. Total group is separated into cases with tumor associated epilepsy (TAE) and no-TAE cases, shown in light gray and dark gray respectively. **(B)** FA values of reciprocal cortico-striatal and cortico-thalamic projections ipsi- and contralateral to tumor separated for TAE and no-TAE cases. Box and whiskers are given, showing the median and 25–75% percentile with whiskers for the minimum and maximum. The ipsilateral hemisphere data is shown in unchecked boxes, checked boxes are contralateral hemisphere. Wilcoxon matched-pairs signed rank tests were used per structure. Asterisks indicate significant results. *P ≤ 0.02.

With regard to the FA value, a significant change was found also in the caudate and thalamus of TAE patients, but not in the no-TAE patient group (Figure 5C and Table 7). The FA values of the cerebellar cortex and WM were not different (Figure 5D). Analysis on the tracts connecting the subcortical regions to the cortex showed a significant decrease in volume of tracts to putamen ipsilateral to the tumor in the TAE group, but not in the tracts to caudate and thalamus, similarly to the analysis of the whole cohort (Figure 6A compared with Figure 4B and Table 8). For the FA values to these structures, no differences between hemispheres were observed (Figure 6B, Table 9). To further explore the effect of epilepsy on these alterations, we plotted the volume and FA ratio's per structure against the seizure frequency, seizure type and tumor grade. No significant relationship between any of these subgroups and the FA and volume alterations was found for both the nuclei and the connecting tracts (see Supplementary Figures 4–6).

**Table 7:** Fractional anisotropy alterations in abbrevviation TAE and no-TAE group in subcortical nuclei and cerebellum.

**Structure**		**FA ipsilateral**	**FA contralateral**	***P***	**Pairing spearman r**	***P***
Caudate	TAE	0.252 ± 0.037	0.234 ± 0.034	<0.001	0.65	<0.001
Caudate	no-TAE	0.2243 ± 0.034	0.236 ± 0.043	0.82	0.62	0.04
Putamen	TAE	0.277 ± 0.033	0.271 ± 0.030	0.03	0.85	<0.001
Putamen	no-TAE	0.259 ± 0.024	0.267 ± 0.023	0.20	0.80	<0.001
Thalamus	TAE	0.331 ± 0.034	0.321 ± 0.030	0.02	0.79	<0.001
Thalamus	no-TAE	0.296 ± 0.028	0.305 ± 0.033	0.82	0.28	0.23
Cerebellar WM	TAE	0.386 ± 0.039	0.387 ± 0.043	0.58	0.92	<0.001
Cerebellar WM	no-TAE	0.367 ± 0.029	0.368 ± 0.028	0.73	0.87	0.002
Cerebellar cortex	TAE	0.206 ± 0.021	0.211 ± 0.021	0.07	0.80	<0.001
Cerebellar cortex	no-TAE	0.208 ± 0.016	0.209 ± 0.012	0.91	0.67	0.03

*Mean FA ± standard deviation for ipsilateral hemisphere in relation to the tumor and the contralateral hemisphere, tumor associated epilepsy (TAE) group n = 38 pairs, no-TAE group n = 19 pairs. For cerebellum: TAE n = 37 pairs, no-TAE group n = 16 pairs. Wilcoxon matched-pairs signed rank test. For each structure the pairing Spearman r with significance is given. WM, white matter; TAE, tumor associated epilepsy*.

**Table 8:** Volume alterations of cortico-striatal and cortico-thalamic tracts in TAE and no-TAE groups.

**Cortical WM tracts to/from**		**Ipsilateral volume (mm^3^)**	**Contralateral volume (mm^3^)**	***P***	**Pairing spearman r**	***P***
Caudate	TAE	31,340 ± 13,190	29,712 ± 11,311	0.13	0.68	<0.001
Caudate	no-TAE	30,228 ± 16,998	34,645 ± 19,683	0.76	0.39	0.11
Putamen	TAE	53,477 ± 18,693	67,291 ± 18,372	<0.001	0.49	<0.001
Putamen	no-TAE	49,230 ± 19,376	70,520 ± 16,665	0.12	−0.1	0.39
Thalamus	TAE	60,972 ± 18,702	59,814 ± 15,661	0.43	0.84	<0.001
Thalamus	no-TAE	65,611 ± 16,938	63,432 ± 21,150	0.36	0.89	<0.001

*Mean volumes ± standard deviation for ipsilateral hemisphere in relation to the tumor and the contralateral hemisphere, tumor associated epilepsy (TAE) group n = 56 pairs, no-TAE group n = 11 pairs. Wilcoxon matched-pairs signed rank test. For each structure the pairing Spearman r with significance is given. WM, white matter, TAE, tumor associated epilepsy*.

**Table 9:** Fractional anisotropy alterations of cortico-striatal and cortico-thalamic tracts in TAE and no-TAE groups.

**Cortical WM tracts to/from**		**FA ipsilateral**	**FA contralateral**	***P***	**Pairing spearman r**	***P***
Caudate	TAE	0.376 ± 0.049	0.375 ± 0.041	0.42	0.53	<0.001
Caudate	no-TAE	0.350 ± 0.037	0.378 ± 0.058	0.37	0.001	0.49
Putamen	TAE	0.416 ± 0.067	0.436 ± 0.039	0.08	0.55	<0.001
Putamen	no-TAE	0.379 ± 0.078	0.411 ± 0.042	0.20	−0.17	0.30
Thalamus	TAE	0.418 ± 0.041	0.411 ± 0.040	0.04	0.75	<0.001
Thalamus	no-TAE	0.412 ± 0.027	0.404 ± 0.027	0.37	0.55	0.04

*Mean FA ± standard deviation for ipsilateral hemisphere in relation to the tumor and the contralateral hemisphere, tumor associated epilepsy (TAE) group n = 56 pairs, no-TAE group n = 11 pairs. Wilcoxon matched-pairs signed rank test. For each structure the pairing Spearman r with significance is given. WM, white matter; TAE, tumor associated epilepsy*.

In conclusion, the structural and connectivity changes in subcortical areas were significantly more prevalent in the patients with TAE. Thus, our data indicate that LGG patients with TAE are more likely to show structural and connectivity aberrations in various distant brain regions, highlighting the potential correlation between epileptogenesis and subcortical structural changes of in LGG patients.

## Discussion

This study reveals new insights on the impact of low grade glioma and tumor associated epilepsy on the structural integrity of the subcortical nuclei and cerebellum, as well as the cortico-striatal and cortico-thalamic connectivity. Using a large DWI dataset exclusively from LGG patients that underwent awake surgery, we found structural alterations ipsilateral from the tumor for both the volume and the DWI parameters of the caudate nucleus, the putamen, and thalamus. We found that the cortico-striatal and cortico-thalamic projections connected to these nuclei ipsilateral to the tumor show altered diffusion values. Furthermore, we explored the correlation between tumor associated epilepsy and these alterations and found that the patients with TAE are prone to structural alterations of the subcortical nuclei.

The volumetric analysis of subcortical nuclei and cerebellum accentuates that on the side of the tumor, the volume of the striatum and thalamus is significantly reduced compared to the contralateral hemisphere and that the cerebellar cortex contralateral to the tumor is affected. This volume reduction suggests that LGG causes damage to the large scale brain networks and that both specific subcortical nuclei and the cerebellum suffer from forms of structural and functional alterations because of this effect. We found no significant correlation between degree of volume and FA change and tumor grade. Although it is possible that faster and more invasively growing tumors have more impact on these measures, we expect only a limited effect of higher grade phenomena such as edema and mass effect on the studied long-distance connections and structures far away from the tumor environment.

Volume decrease of subcortical structures has been described previously in other brain diseases such as temporal lobe epilepsy (27, 43, 44) on the side of the focus, generalized tonic-clonic seizure epilepsy (29), juvenile myoclonic epilepsy (45), but also in psychiatric disease such as schizophrenia (46) and even in chronic stroke patients (47). Inter-patient hemispheric differences in diffusion parameters and volume should be considered, but these effects are eliminated in this analysis by the in-patient comparison with the contralateral hemisphere and the presence of both right-sided and left-sided tumors in the database. Interestingly, our analysis indicates that the cerebellum, a region far removed from the initial tumor site but highly connected through the subcortical nuclei, also suffers from volume reduction in the cortex of the contralateral cerebellar hemisphere. Related to this finding, a recent report performing resting state functional MRI (rs-fMRI) in awake surgery for low grade glioma also demonstrates a hypometabolism occurring in the contralesional cerebellar hemisphere directly post-surgery lasting for several months after surgery (48). Given our results, it is possible that a change in structural connectivity occurs already before surgery and affects these large-scale brain networks. An important additional question is then if these observed changes remain present after resection and additional treatment of the glioma; this will have to be determined by post-surgical MRI scans both directly after surgery and during follow-up.

To calibrate our results to a healthy population, we used 60 patients from the Human Connectome Project (http://www.humanconnectomeproject.org). As a control measure for the found volumetric differences, we determined if there were any volumetric left-right differences between the subcortical nuclei, cerebellum and the cortico-striatal and cortico-thalamic tracts in healthy subjects. For the subcortical nuclei, we found a larger volume of the left thalamus compared to right, for caudate and putamen no significant volumetric differences were found. In cerebellum the right cerebellar cortical volume was significantly enlarged compared to the left, no difference in white matter volume was observed (Supplementary Figure 7). What is important to consider when interpreting this data however, is that these scans are from a young population of patients (all under 30 years old) and that the found differences are between the left and right hemisphere. Patients with tumor located at both left and right sides were included in this study. In our analysis we compared structures ipsilateral to the tumor to the contralateral side, which makes direct comparison with left/right ratio unfeasible. Furthermore, data from other studies comparing left and right nuclei have found either no difference (49–51) or a larger volume of structures in the left hemisphere (52, 53).

The cortico-striatal and cortico-thalamic tract analysis resulted in a notable reduction in the volume of the tracts to/from putamen vs. the tracts to/from caudate and thalamus, which both seemed to be a lot less affected. One attributing factor to this finding could be the anatomical location of the putamen as the most lateral of the subcortical nuclei, being most susceptible to changes caused by the tumor. Furthermore, the functional location of the putamen as the recipient of many projections from the primary motor and supplementary motor cortex combined with this specific database with many tumors located around these eloquent regions could lead to many fibers to putamen being affected. In contrast, DWI analysis showed that the FA and MD values, both indicative of the consistency of tracts, were selectively altered in the thalamic tracts, despite the unaltered thalamic tract volume. The cause for the change in FA and MD values remain unknown. Nevertheless, a sharp contrast in the specific alterations between the putamen tracts and thalamus tracts suggests complex cellular and circuit mechanisms that causes these deficits.

Given that the volumetric and diffusion alterations remained significant in the subgroup of cases with TAE and were not significant in the patients without TAE, our research suggests a tight relationship between TAE and structural alternations. A correlation between epilepsy and structural alterations has been reported in patients with temporal lobe epilepsy. These alterations are found both inside the temporal lobe and in extra-temporal structures including the basal ganglia and thalamus and even extending into the cerebellum (27–29). A recent study also illustrated that the spread of WM abnormalities correlates with the occurrence of neurocognitive deficits (30). Yet, our data does not warrant a causal relation between TAE and structural alterations in LGG patients. Future study aiming at illustrating the cause and consequences of TAE will be extremely important. Another interesting phenomenon warrants careful interpretation since the observed changes in FA and MD especially do not seem to match the findings in DWI studies of primary forms of epilepsy. We found that FA, which measures the directionality of water diffusion within a voxel, ipsilateral to the side of the tumor was increased in both the subcortical nuclei and the WM pathways connecting the cortex to the nuclei. The MD values decreased ipsilateral to the tumor. This is in contrast to studies in temporal lobe epilepsy with mesial temporal sclerosis, which demonstrate a decrease in FA and a decrease in MD values on the side of the focus (30, 54, 55). It therefore is possible that the mechanism underlying these findings, even though they appear to be strongly mediated by the presence of tumor associated epilepsy, is very different from the alterations seen in primary epilepsies.

It remains a question what kind of cellular alterations the FA represents. FA is determined by a combination of axonal density, caliber, and degree of myelination, but is also determined by other processes such as microglial function, inflammation and tissue architecture (56, 57). The possible causes for the observed microstructural changes with increased FA and decreased MD must be sought in our current understanding of the pathophysiological processes underlying glioma growth. Other studies using DWI to study subcortical gray matter and cerebellum in glioma patients are scarce. Miller and colleagues found in a group of low grade glioma patients that FA was markedly increased in some brain areas further from the tumor (58), which was considered a consequence of WM compression. Studies using DWI to investigate both glioma and TAE are even more scarce, however Wieshmann et al. did find an association between oligodendroglioma proximity to the genu of the corpus callosum and the risk of developing generalized seizures over focal seizures (59).

Presently, oligodendrocyte precursor cells (OPCs) are being viewed with much interest in relation to their role in tumor etiology and are being proposed as a major cell of origin for glioma tumorigenesis (60, 61). It has recently been shown that glioma cells generate mitogenic signals to neuronal and oligodendroglial precursor cells and that they use the endogenous neuronal activity and secreted mitogens to promote their own survival and proliferation (62, 63). The proliferation and transformation of OPCs for tumor-induction purposes is a long and intricate process during which local neuronal networks and the microenvironment are likely to be severely affected and altered. Myelin forming cells in the brain are plastic and sensitive to activity-dependent clues, adapting the myelination of neurons based on the needs of the environment (64). In glioma, tumor cells are able to induce activation of surrounding neurons which leads a situation where higher activity is favored, resulting in a potential trigger for OPC proliferation and differentiation and oligodendrocyte activation, leading to increased myelination of activated axons. What is even more intriguing in the perspective of glioma and TAE is the finding that initial induction of myelin synthesis is regulated within the OPC process that is driven by synaptic vesicular glutamate release (65). Recent work on the pathophysiology of tumor associated epilepsy in glioma has indicated the x${}_{\text{c}}^{-}$ system as an important survival and proliferation system that is used by glioma cells, leading to the simultaneous uptake of cysteine into the glioma cell and the excretion of intracellular glutamate into the peri-cellular space (66). This creates a high glutamate concentration in the synaptic cleft as well as the surrounding peri-tumoral cortex, leading to the induction of hyper-excitation of peri-tumoral neurons and eventually to the development of glioma induced epileptic seizures (66–68). Based on the previous, the combination of glioma cells and activated neurons can in turn trigger OPC differentiation and signal the oligodendrocytes to increase the myelination of active axons. These findings would support the observed preservation of all alterations in the cases with epilepsy, whereas the alterations were no longer significant in cases without epilepsy.

What remains first, is the question whether the observed volumetric and diffusion changes are a consequence directly of glioma growth or that they occur due to the subsequent development of TAE. Based on the information in this cohort, we are not able to unambiguously address this question. Secondly, we were not able with this database to show a significant correlation with seizure frequency, type or a difference in tumor grade. This could imply that the observed changes are unrelated to these factors but most likely the power of our dataset is still insufficient to address this with enough detail. Therefore, new studies are needed to study these long-range connections with a larger group of patients with and without TAE. Especially, studies are needed that focus on combining long-term neurocognitive testing pre- and post-surgery with structural DWI analysis. Longitudinal MRI studies following the progression of tumor genesis would allow a careful dissection of the causal relation between structural deficits and TAE. Also, it is necessary to investigate that when seizures are resolved after successful surgery, if the diffusion and volumetric alterations remain, and if any values change when seizures start again indicating tumor recurrence.

A pre-existing volume loss in subcortical nuclei and connecting WM tracts could also render the brain more vulnerable to resection of cortex so that it has less reserve to compensate for cortical injury during surgery. These questions again require long-term imaging and neurocognitive testing to determine if there are differences in immediate and long-term outcomes for patients with significant volume loss pre-surgery. In this retrospective cohort we did not have neurocognitive testing data available for these specific tracts to correlate our findings to neuro-cognitive performance. Recently however, using patients from the same cohort, a study was published on white matter tracts involved in language and attention showing a significant relationship between WM tract alterations and language and attention performance (69). In the future, an important question will be if the degree of subcortical volume loss correlates to the severity of neurocognitive deficits, our current database however lacks the outcome data to address this question.

Lastly, in light of the ongoing discussion about specifically locating and resecting the epileptic focus during glioma surgery (70, 71), more studies are also needed to determine the effect of removing the epileptic focus on network structural integrity in this patient group.

## Conclusion

Based on our findings from volumetric and diffusion analysis in a cohort of low grade glioma patients, glioma has a far-reaching impact on subcortical gray matter nuclei and the cerebellum, leading to changes in both volume and diffusion parameters. Tumor associated epilepsy due to the presence of a LGG is most likely an important contributing factor to the observed alterations, although its magnitude cannot be concluded based on this data. Our findings support the hypothesis that glioma can lead to impaired neuro-cognitive functioning preoperatively by affecting large-scale neuronal networks and altering subcortical nuclei and cerebellar regions.

## Ethics statement

The study was approved by the medical ethical committee of Erasmus MC–University Medical Center, which waived the need for written informed consent from the patients because of the retrospective nature of the study and the (emotional) burden that would result from contacting the patients or their relatives to obtain consent.

## Author contributions

BB, KP, ZG, and AV conceptualized and designed the study. BB, KP, and MS guided the data collection. BB and KP analyzed the data. BB and ZG drafted and revised the study with intellectual inputs from KP, AV, MS and CD.

### Conflict of interest statement

The authors declare that the research was conducted in the absence of any commercial or financial relationships that could be construed as a potential conflict of interest.
