# Supplementary material for: The Impacts of Tumor and Tumor Associated Epilepsy on Subcortical Brain Structures and Long Distance Connectivity in Patients With Low Grade Glioma
Source: Front Neurol. 2018 Nov 27;9:1004. doi: 10.3389/fneur.2018.01004 (PMC6277571; doi:10.3389/fneur.2018.01004)
Supplement: Supplementary file 1 [file Data_Sheet_1.docx]

Supplementary Material

**The impacts of tumor and tumor associated epilepsy on subcortical brain structures and long distance connectivity in patients with low grade glioma**

Bibi L.J. Bouwen^1,2,#^, Kay J. Pieterman^3,#^, Marion Smits^3^, Clemens M.F. Dirven^2^, Zhenyu Gao^1,$,*^ and Arnaud J.P.E. Vincent^2,$^

1. Department of Neuroscience, Erasmus MC, University Medical Center Rotterdam, The Netherlands
2. Department of Neurosurgery, Erasmus MC, University Medical Center Rotterdam, The Netherlands
3. Department of Radiology and Nuclear Medicine, Erasmus MC, University Medical Center Rotterdam, The Netherlands

# Both authors contributed equally to this work

$ Both authors co-supervised this project

* Correspondence

Dr. Zhenyu Gao [z.gao@erasmusmc.nl](mailto:z.gao@erasmusmc.nl)

# Supplementary Figures

**
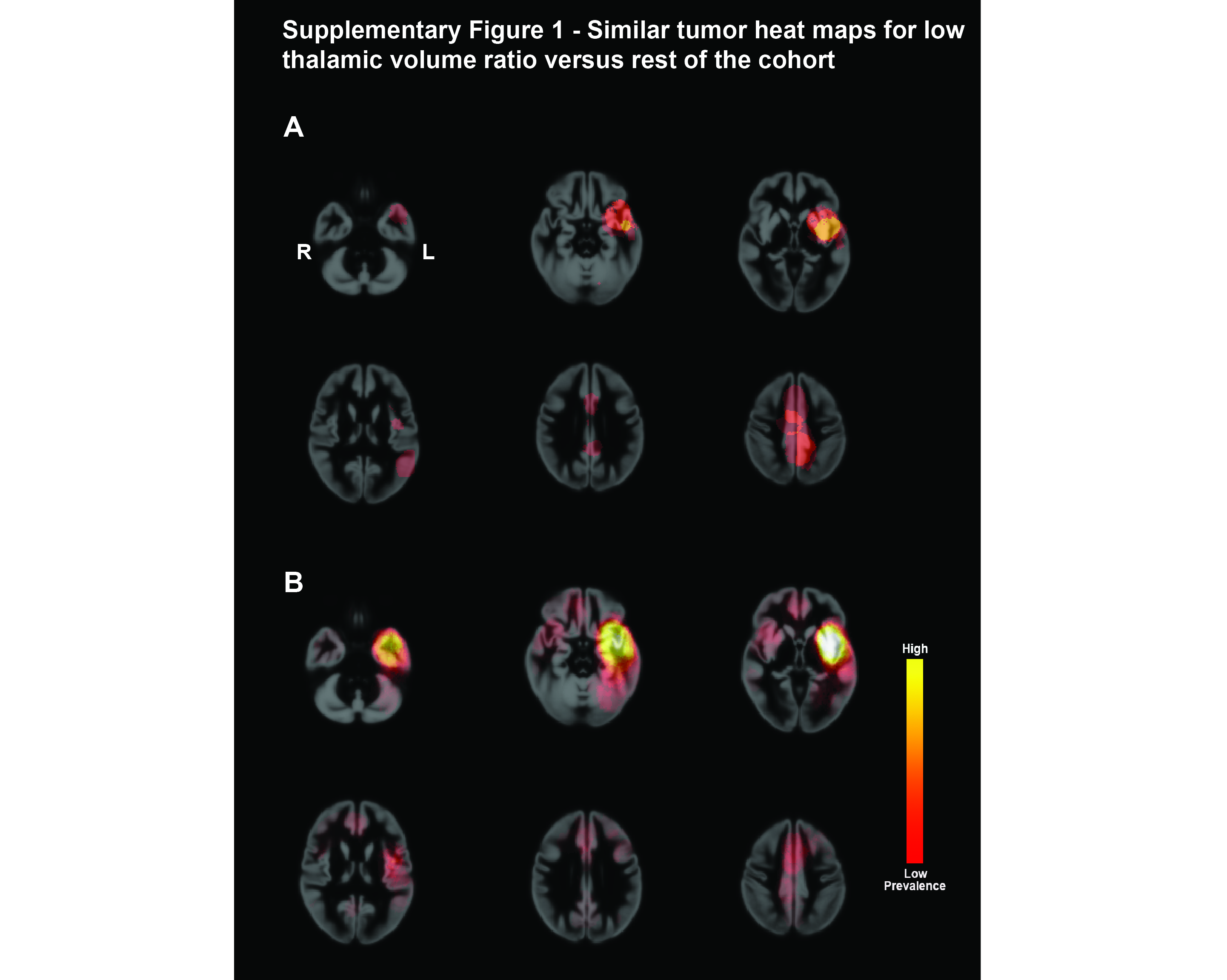
**

**Supplementary Figure 1 – Similar tumor heat maps for low thalamic volume ratios versus rest of the cohort**

(A) Heat maps projected on smoothed transverse cortical template images showing tumor masks from the patients with very low thalamic volume ratios (N=13). (B) Heat maps of tumors from the rest of the cohort (N=44). Differences in colors occur due to higher and lower prevalence. Color key for heat map shown on the far right.

**
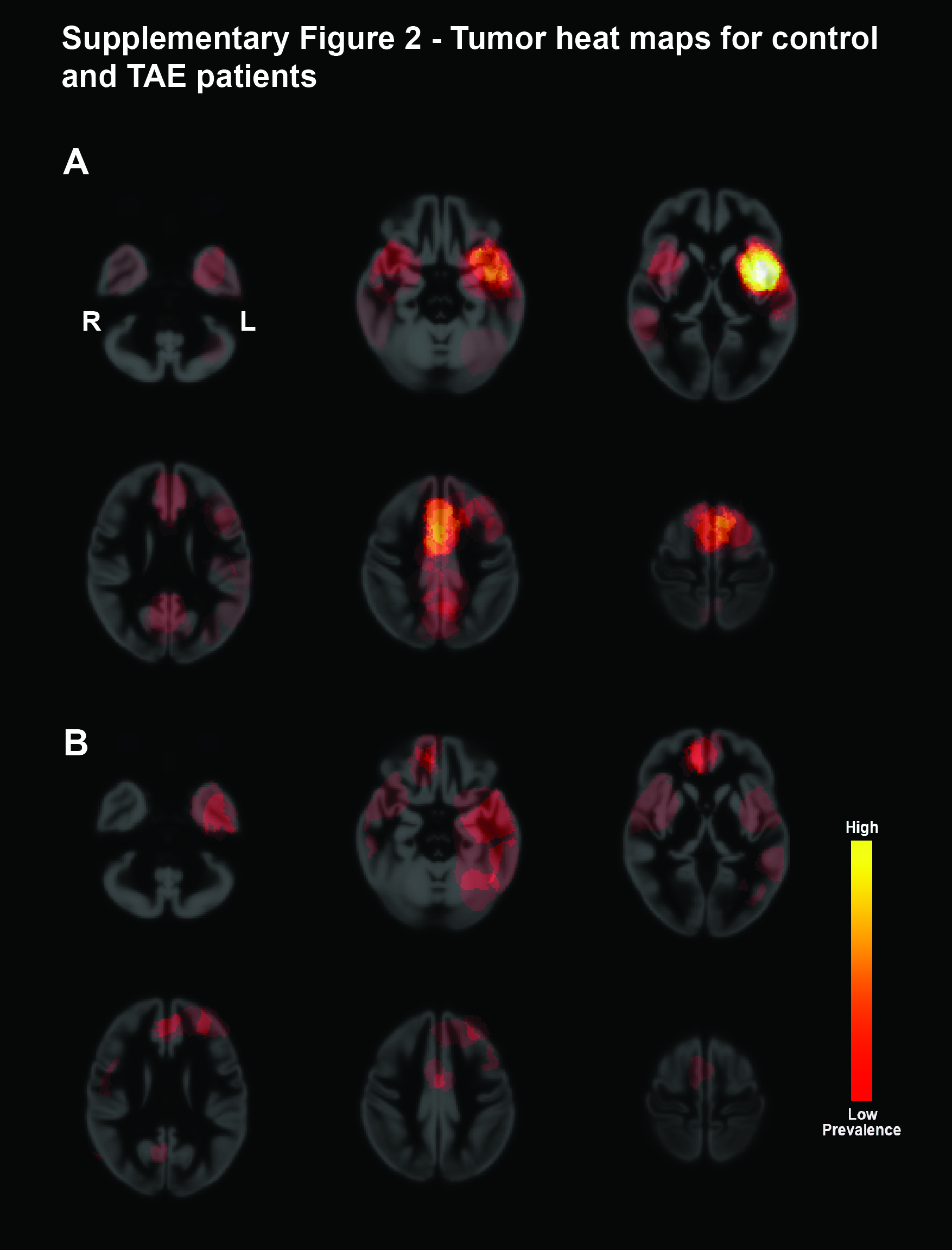
**

**Supplementary Figure 2 – Tumor heat maps for control and TAE patients**

(A) Heat maps projected on smoothed transverse cortical template images showing tumor masks from all patients with tumor associated epilepsy (N=38). (B) Heat maps of tumors from no-TAE patients (N=19). Differences in colors occur due to higher and lower prevalence. Color key for heat map shown on the far right.

**
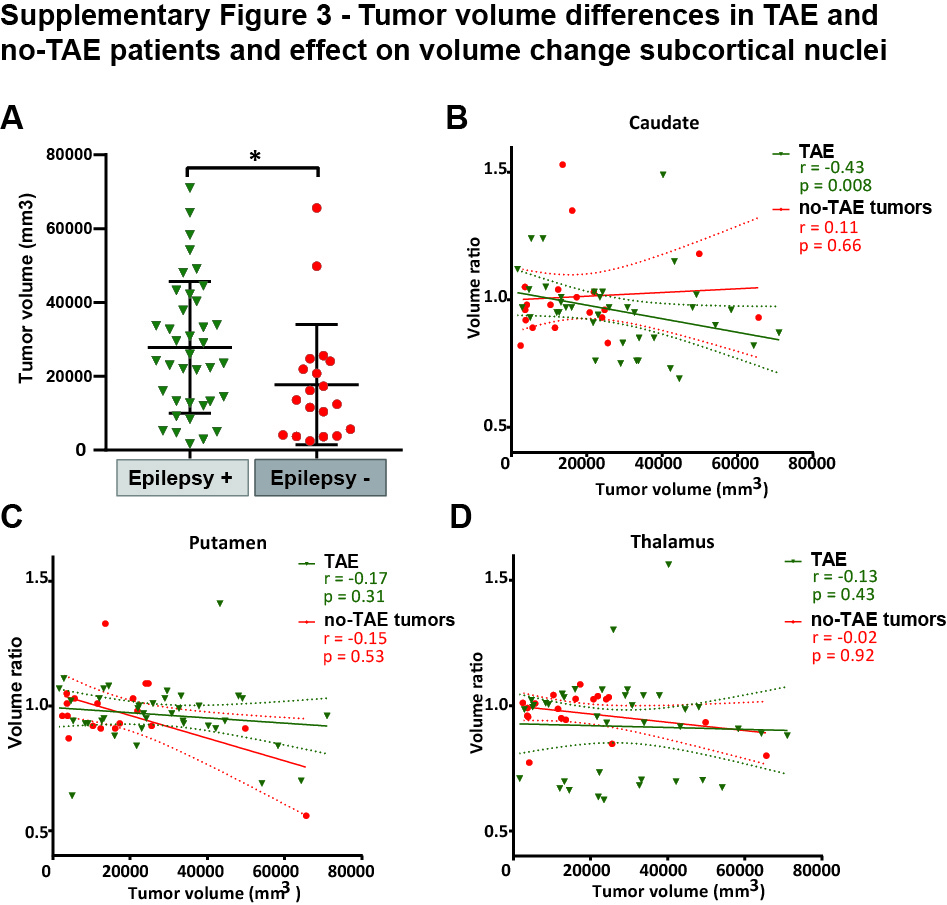
**

**Supplementary Figure 3 – Tumor volume differences in TAE and no-TAE patients**

(A) Tumor volumes in mm^3^ of TAE patients (red) and no-TAE patients (blue). Box and whiskers are given, showing the median and 25%-75% percentile with whiskers for the minimum and maximum. In (B-D) the correlation between tumor volume and volume ratio (ipsilateral volume divided by contralateral volume) is given for caudate, putamen and thalamus respectively. Mann Whitney two-tailed test was used to compare groups. The data is split for TAE (green) and no-TAE patients (red). Linear regression line with 95% confidence interval shown per group. Spearman r and p-value are given.

**
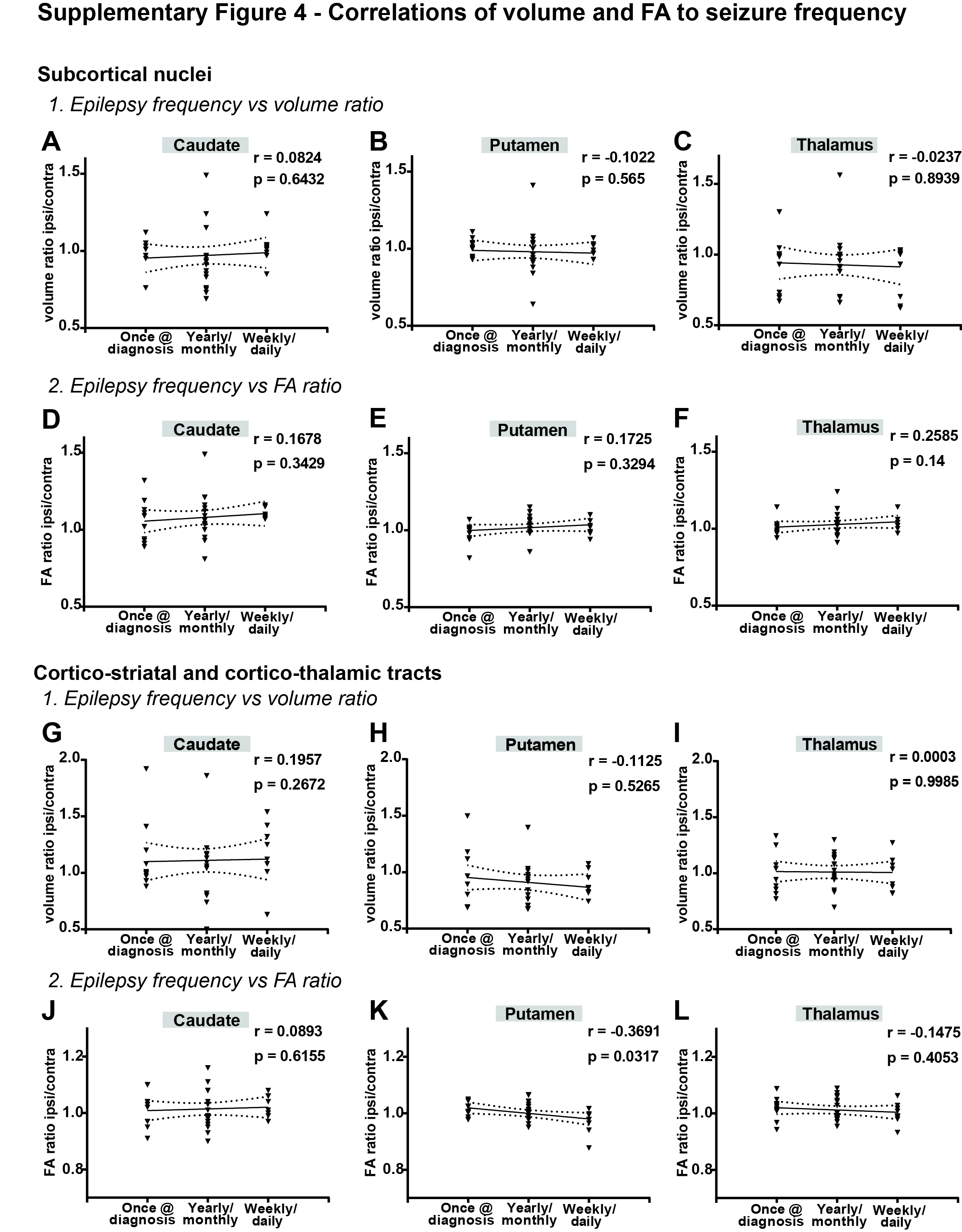
**

**Supplementary Figure 4 – Correlation of volume and FA ratios to seizure frequency**

Correlations of seizure frequency to volume ratio (1) and FA ratio (2) (ratios calculated by dividing ipsilateral value by contralateral value) for both the subcortical nuclei and the cortico-striatal and cortico-thalamic tracts. (A-C) Correlation plots of seizure frequency to volume ratio in caudate, putamen and thalamus. (D-F) Correlation plots of seizure frequency to FA ratio in caudate, putamen and thalamus. (G-I) Correlation plots of seizure frequency to volume ratio of reciprocal cortico-caudate, cortico-putamen, and cortico-thalamic tracts. (J-L) Correlation plots of seizure frequency to FA ratio of reciprocal cortico-caudate, cortico-putamen, and cortico-thalamic tracts. Individual values and linear regression line with 95% confidence interval are displayed. Spearman r and p-value are given for each plot.

**
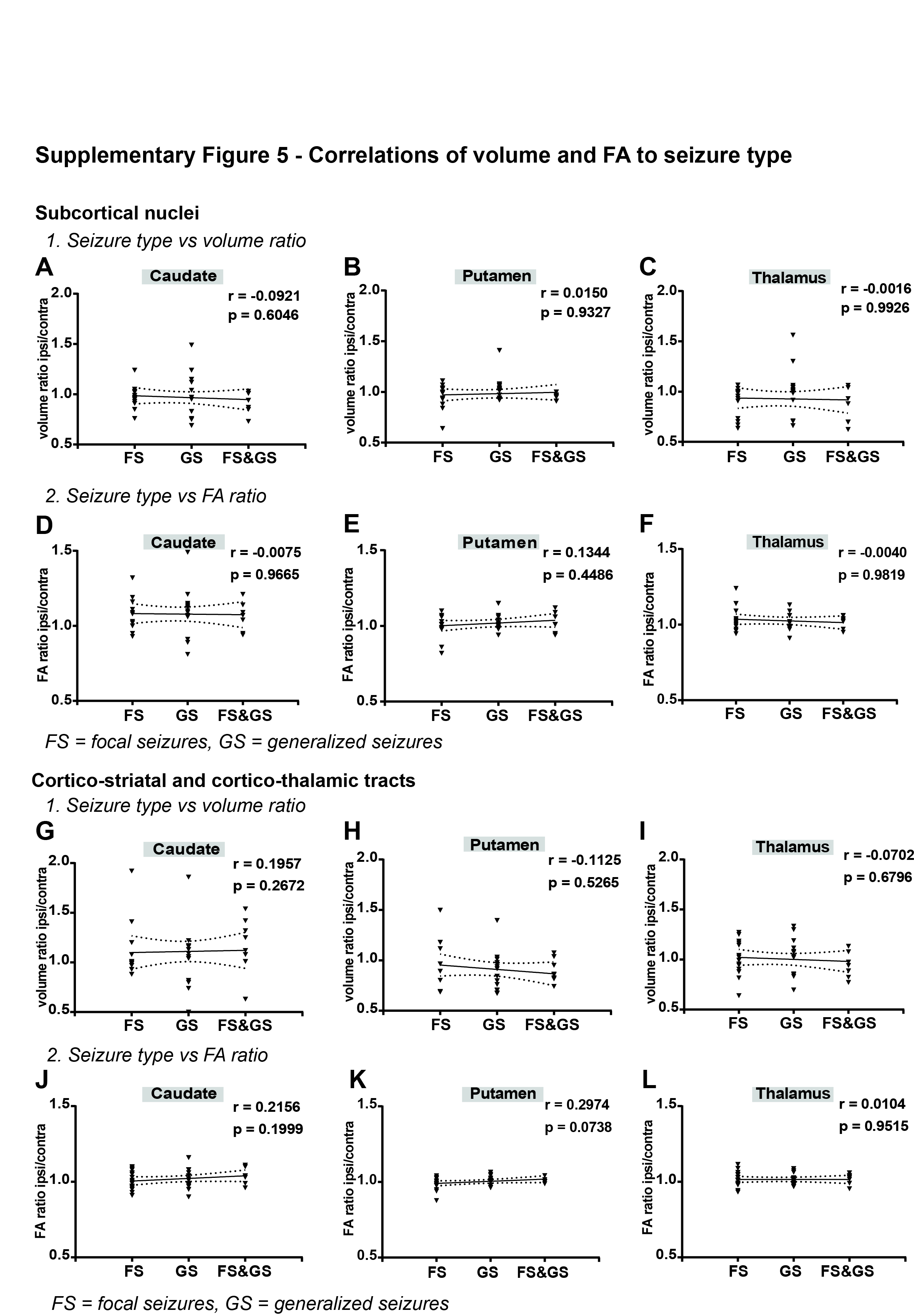
**

**Supplementary Figure 5 – Correlation of volume and FA ratios to seizure type**

Correlations of seizure type to volume ratio (1) and FA ratio (2) (ratios calculated by dividing ipsilateral value by contralateral value) for both the subcortical nuclei and the cortico-striatal and cortico-thalamic tracts. (A-C) Correlation plots of seizure type to volume ratio in caudate, putamen and thalamus. (D-F) Correlation plots of seizure type to FA ratio in caudate, putamen and thalamus. (G-I) Correlation plots of seizure type to volume ratio of reciprocal cortico-caudate, cortico-putamen, and cortico-thalamic tracts. (J-L) Correlation plots of seizure type to FA ratio of reciprocal cortico-caudate, cortico-putamen, and cortico-thalamic tracts. Individual values and linear regression line with 95% confidence interval are displayed. Spearman r and p-value are given for each plot.

**
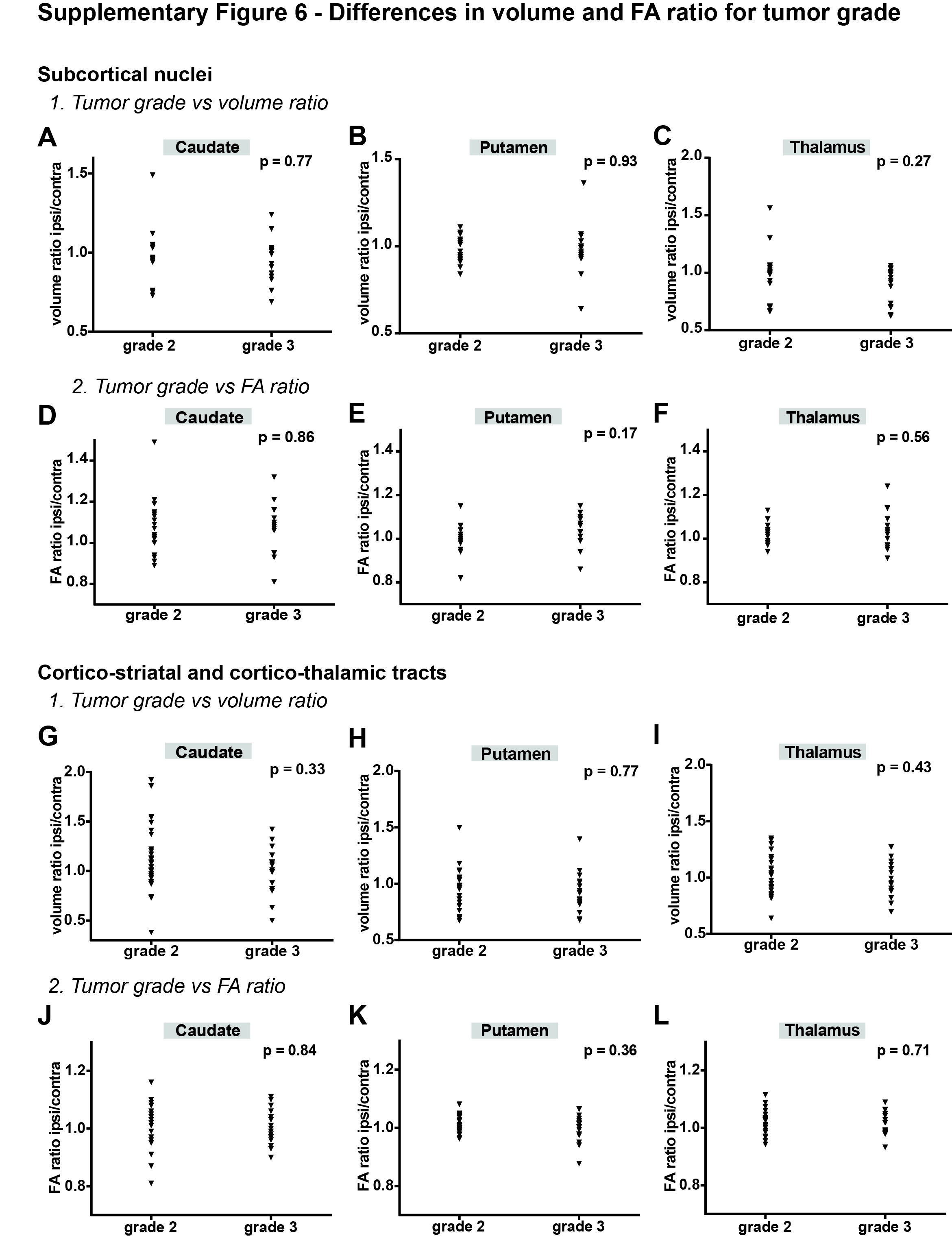
**

**Supplementary Figure 6 – Correlation of volume and FA ratios to tumor grade**

Comparison of volume ratio (1) and FA ratio (2) differences by tumor grade (ratios calculated by dividing ipsilateral value by contralateral value) for both the subcortical nuclei and the cortico-striatal and cortico-thalamic tracts. (A-C) Comparison of volume ratios in grade 2 and 3 tumors in caudate, putamen and thalamus. (D-F) Comparison of FA ratios in grade 2 and 3 tumors in caudate, putamen and thalamus. (G-I) Comparison of volume ratio of reciprocal cortico-caudate, cortico-putamen, and cortico-thalamic tracts in grade 2 and 3 tumors. (J-L) Comparison of FA ratio of reciprocal cortico-caudate, cortico-putamen, and cortico-thalamic tracts in grade 2 and 3 tumors. Individual values are displayed. Student’s T-test was used to compare groups, P-values are given for each plot.

**
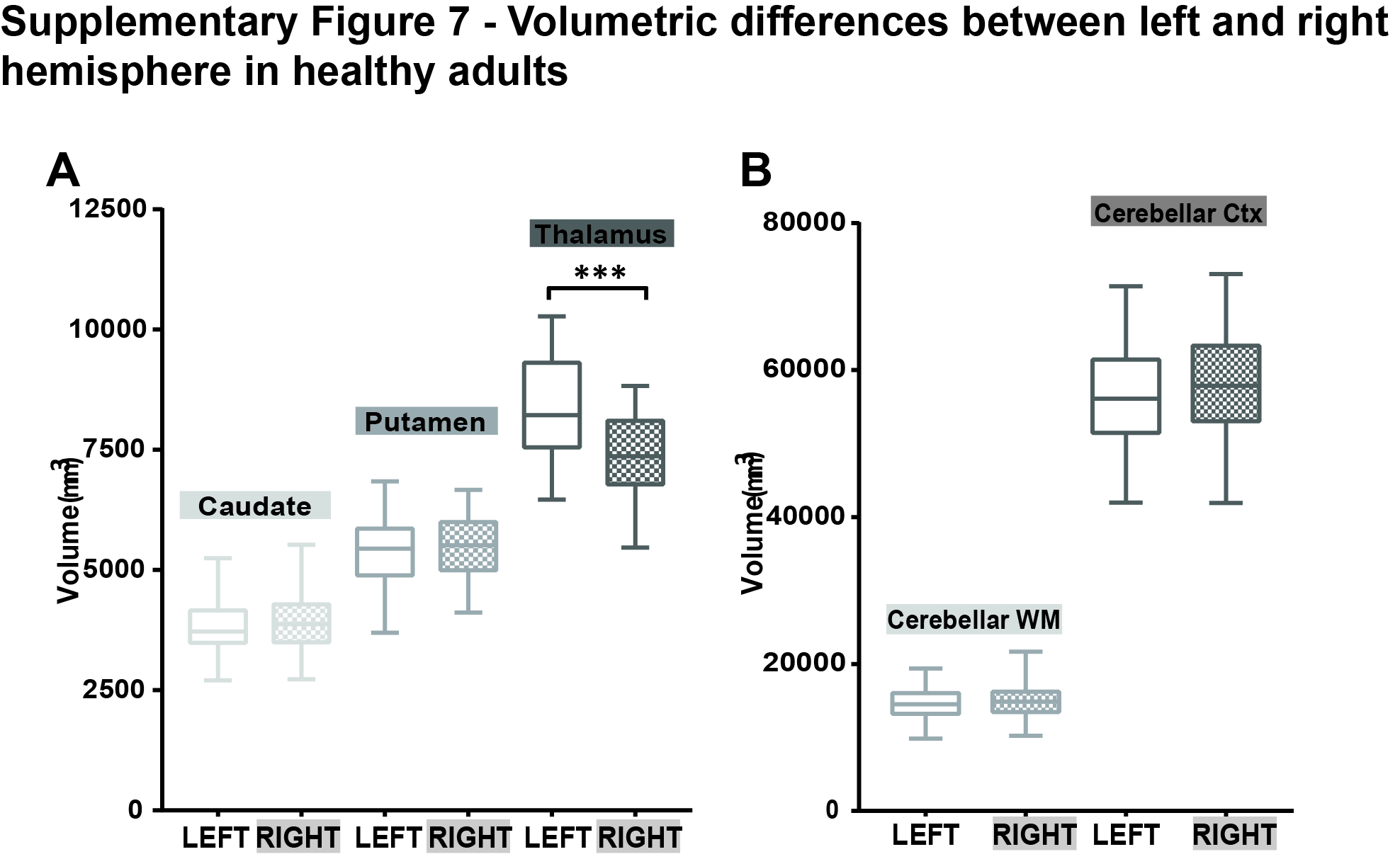
**

**Supplementary Figure 7 – Volumetric differences between left and right hemisphere in healthy adults**

(A) Volumes of left and right subcortical nuclei caudate, putamen and thalamus are compared. Box and whiskers are given showing the median and 25%-75% percentile with whiskers for the minimum and maximum. The left hemisphere data is shown in unchecked boxes, checked boxes are right hemisphere volumes. Volume is given in mm^3^. In (B) the volumes for cerebellar WM and cortex are shown. Mann-whitney test used to compare left and right hemisphere for each structure. Asterisks indicate significant results. *** P<0.001. WM = white matter, Ctx = cortex.

**
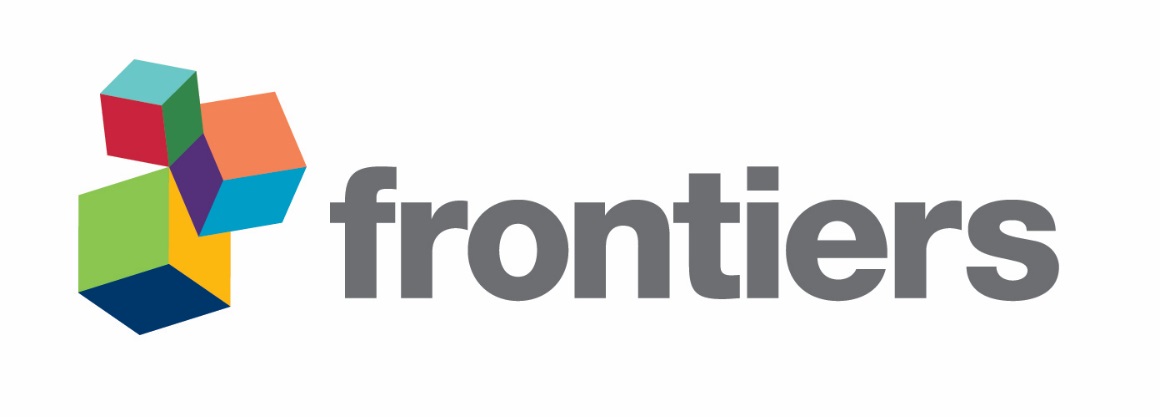
**
